# miR-874: An Important Regulator in Human Diseases

**DOI:** 10.3389/fcell.2022.784968

**Published:** 2022-04-06

**Authors:** Qiudan Zhang, Chenming Zhong, Qianqian Yan, Ling-hui Zeng, Wei Gao, Shiwei Duan

**Affiliations:** ^1^ School of Medicine, Zhejiang University City College, Hangzhou, China; ^2^ Medical Genetics Center, School of Medicine, Ningbo University, Ningbo, China

**Keywords:** miR-874, cancer, tumor suppressor, non-cancer, diagnosis, prognosis, ceRNA, pathway

## Abstract

miR-874 is located at 5q31.2, which is frequently deleted in cancer. miR-874 is downregulated in 22 types of cancers and aberrantly expressed in 18 types of non-cancer diseases. The dysfunction of miR-874 is not only closely related to the diagnosis and prognosis of tumor patients but also plays an important role in the efficacy of tumor chemotherapy drugs. miR-874 participates in the ceRNA network of long non-coding RNAs or circular RNAs, which is closely related to the occurrence and development of cancer and other non-cancer diseases. In addition, miR-874 is also involved in the regulation of multiple signaling pathways, including the Wnt/β-catenin signaling pathway, Hippo signaling pathway, PI3K/AKT signaling pathway, JAK/STAT signaling pathway, and Hedgehog signaling pathway. This review summarizes the molecular functions of miR-874 in the biological processes of tumor cell survival, apoptosis, differentiation, and tumorigenesis, and reveal the value of miR-874 as a cancer biomarker in tumor diagnosis and prognosis. Future work is necessary to explore the potential clinical application of miR-874 in chemotherapy resistance.

## Background

MicroRNA (miRNA) is a short RNA of 19–25 nucleotides in length that can regulate the post-transcriptional silencing of target genes. A single miRNA can target hundreds of mRNAs, thereby affecting the signal transduction of multiple pathways (Lu and Rothenberg, 2018). miRNA expression profiling shows that abnormal miRNA expression is related to the occurrence and progression of tumors and the response to anticancer drugs (Iorio and Croce, 2017). In addition, almost all developmental, physiological, and disease-related processes seem to be linked to miRNAs (Sun and Lai, 2013).

**FIGURE 1:**
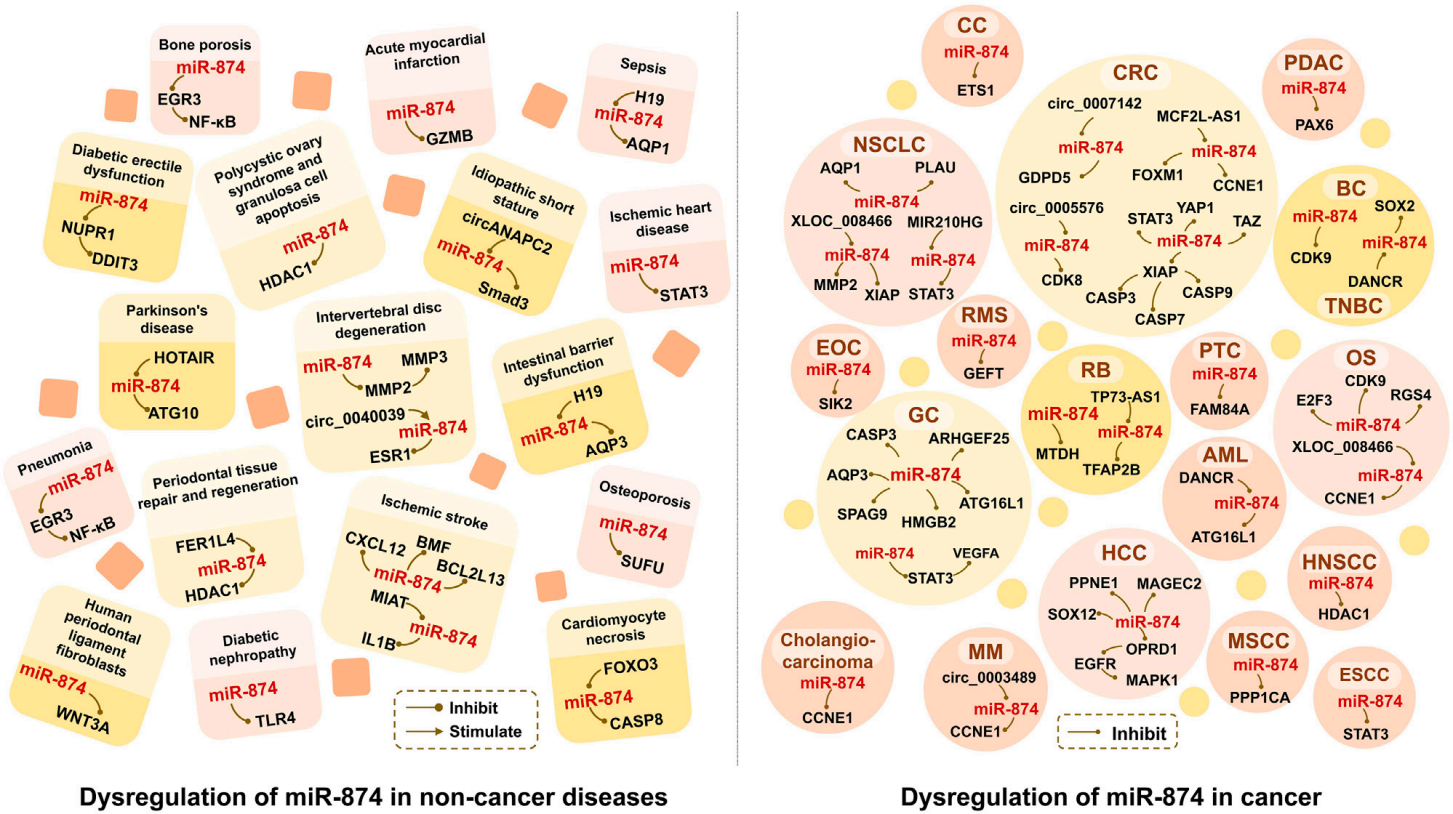
Dysregulation of miR-874 in human cancer and non-cancer diseases. HCC, Hepatocellular carcinoma; PDAC, Pancreatic ductal adenocarcinoma; NSCLC, Non-small cell lung cancer; OS, Osteosarcoma; EOC, Epithelial ovarian cancer; CC, Cervical cancer; TNBC, Triple negative breast cancer; r BC, Breast cancer; CRC, Colorectal cancer; ESCC, Esophageal squamous cell Squamous cell carcinoma; MSCC, Maxillary sinus squamous cell carcinoma; RB, Retinoblastoma; GC, Gastric cancer; RMS, Rhabdomyosarcoma; PTC, Papillary thyroid carcinoma; MM, myeloma; AML, acute myeloid leukemia; HNSCC, head and neck squamous cell carcinoma.

microRNA-874 (miR-874) is a new anti-cancer miRNA (Zhang et al., 2018b). It was originally obtained by sequencing a small RNA library (Song et al., 2016) and was first characterized in normal human cervical tissue samples (Xia et al., 2018). miR-874 is located on chromosome 5q31.2 that is often deleted in cancer (Zhang et al., 2018b). There are many target genes of miR-874 (Figure 1). By inhibiting the expression of these target genes, miR-874 is widely involved in cell proliferation, apoptosis, invasion, migration, cell cycle, epithelial-mesenchymal transition (EMT), and other cellular processes.

Since there is no comprehensive introduction of miR-874, this work summarizes the abnormal expression of miR-874 in cancer and non-cancerous diseases, and outlines the molecular mechanisms between it and protein-coding genes and non-coding RNAs.

## The Aberrant Expression of miR-874 in Human Diseases

As shown in Table 1, miR-874 is downregulated in at least 22 human malignancies, such as colorectal cancer (CRC) (Huang et al., 2020a; Yu et al., 2020; Que et al., 2017; Zhao and Dong, 2016; Han et al., 2016; Wang et al., 2021; Zhang et al., 2021c), gastric cancer (GC) (Huang et al., 2018; Sun et al., 2020; Yuan et al., 2020; Liu et al., 2017; Zhang et al., 2015; Jiang et al., 2014), hepatocellular carcinoma (HCC) (Zhang et al., 2018b; Jiang et al., 2017; Leong et al., 2017; Song et al., 2016), esophageal squamous cell carcinoma (ESCC) (Yuan et al., 2018), pancreatic ductal adenocarcinoma (PDAC) (Diao et al., 2018), non-small cell lung cancer (NSCLC) (Bu et al., 2020; Yang et al., 2017; Ahmad et al., 2015; Kesanakurti et al., 2013; Wang et al., 2020b), osteosarcoma (OS) (Tang et al., 2018; Liu et al., 2020; Dong et al., 2016; Ghosh et al., 2017), epithelial ovarian cancer (EOC) (Xia et al., 2018) cervical cancer (CC) (Liao et al., 2018; Liu et al., 2021a), retinoblastoma (RB) (Zhang et al., 2018a; Wang et al., 2020a), prostate cancer (PCa) (Pashaei et al., 2017) maxillary sinus squamous cell carcinoma (MSCC) (Nohata et al., 2011), breast cancer (BC) (Li et al., 2020; Zhang et al., 2017; Wang et al., 2014), triple-negative breast cancer (TNBC) (Wu et al., 2020), rhabdomyosarcoma (RMS) (Shang et al., 2019), papillary thyroid carcinoma (PTC) (Ding et al., 2021), endometrial cancer (Witek et al., 2021), cholangiocarcinoma (Pan et al., 2021), glioma (Li et al., 2021a), myeloma (MM) (Tian et al., 2021), head and neck squamous cell carcinoma (HNSCC) (Nohata et al., 2013) and acute myeloid leukemia (AML) (Zhang et al., 2021a).

**TABLE 1:** The role of miR-874 in various cancers.

Cancer Type	The expression of miR-874	Effect *in vivo*	Effect *in vitro*	Regulatory mechanism	References
CRC	downregulation	tumor growth↓	proliferation↓, apoptosis↑	circ_0007142/miR-874-3p/GDPD5	Wang et al. (2021)
	downregulation	tumor growth↓	proliferation↓, migration↓, invasion↓	lncRNA MCF2L-AS1/miRNA-874-3p/FOXM1	Zhang et al. (2021c)
	downregulation	tumor growth↓	proliferation↓, migration ↓, invasion↓, EMT↓	lncRNA MCF2L-AS1/miRNA-874-3p/CCNE1	Huang et al. (2020a)
	downregulation	tumor growth↓	proliferation↓, apoptosis↑	circ_0005576/miRNA-874-3p/CDK8/Wnt/β-catenin	Yu et al. (2020)
	downregulation	tumor growth↓	chemosensitivity↓	miR-874-3p/YAP1, TAZ/Hippo signaling	Que et al. (2017)
	downregulation	tumor growth↓	cell growth↓, apoptosis↑	miR-874-3p/STAT3	Zhao and Dong, (2016)
	downregulation	tumor growth↓	proliferation↓, colony formation↓, apoptosis↑, chemosensitivity↓	miR-874-3p/XIAP/CASP3, CASP7, CASP9/5-FU	Han et al. (2016)
GC	downregulation	prognosis↑	autophagy↓, chemosensitivity↓	miR-874-3p/ATG16L1	Huang et al. (2018)
				
	downregulation	tumor growth↓	proliferation↓, apoptosis↑	miR-874-3p/SPAG9	Sun et al. (2020)
	downregulation	tumor growth↓	proliferation↓, migration↓, invasion↓, EMT↓	IIA (TSN)/miR-874-3p/HMGB2/Wnt/β-catenin	Yuan et al. (2020)
	downregulation	tumor growth ↓	migration ↓, invasion↓, proliferation↓, angiogenesis↓	miR-874/ARHGEF25	Huang et al. (2018)
	downregulation	tumor growth↓ angiogenesis↓	tube formation↓, proliferation↓, migration↓, invasion↓	miR-874-3p/STAT3/VEGFA	Zhang et al. (2015)
	downregulation	tumor growth↓	proliferation↓, colony formation↓, migration↓, invasion↓	miR-874-3p/CASP3, AQP3	Jiang et al. (2014)
HCC	downregulation	tumor growth↓	proliferation↓, migration↓, invasion↓, EMT↓	miR-874/OPRD1/EGFR/MAPK1 caggca	Zhang et al. (2018b)
	downregulation	tumor growth↓	migration↓, invasion↓, EMT↓	miR-874-3p/SOX12	Jiang et al. (2017)
	downregulation	tumour growth↓	cell growth ↓, colony formation↓, apoptosis↑	miR-874-3p/PPNE1	Leong et al. (2017)
	downregulation	tumor growth ↓	proliferation↓, invasion↓	miR-874-3p/MAGEC2	Song et al. (2016)
ESCC	downregulation	tumor growth↓ prognosis↑	proliferation↓, migration↓, invasion↓	miR-874-3p/STAT3	Yuan et al. (2018)
PDAC	downregulation	tumor growth↓	proliferation↓, migration↓, invasion↓	miR-874-3p/PAX6	Diao et al. (2018)
NSCLC	downregulation	tumor growth↓ xenograft growth↓	proliferation↓, migration↓, invasion↓, EMT↓	miR-874-3p/AQP1	Wang et al. (2020b)
		tumor growth↓	proliferation↓, migration↓, invasion↓	MIR210HG/miRNA-874-3p/STAT3	Bu et al. (2020)
	downregulation	tumor growth↓	proliferation ↓, invasion↓, apoptosis↑	lncRNA XLOC_008466/miR-874-3p/MMP2, XIAP	Yang et al. (2017)
	downregulation	invasion↓		CDF/miR-874/MMP2 25901198	Ahmad et al. (2015)
	downregulation	tumor growth ↓	invasion↓, de-differentiation↑, migration↓	miR-874-3p/MMP2, PLAU	Kesanakurti et al. (2013)
OS	downregulation	tumor growth↓	proliferation↓, migration↓, invasion↓	miR-874-3p/CDK9	Tang et al. (2018)
	downregulation	tumor growth↓	proliferation↓, migration↓, invasion↓	miR-874-3p/RGS4	Liu et al. (2020)
	downregulation	tumor growth ↓ metastasis ↓	proliferation↓, apoptosis↑, migration↓, invasion↓	miR-874-3p/E2F3	Dong et al. (2016)
	downregulation	tumor growth↓	invasion↓, migration↓	lncRNA XLOC_008466/miR-874-3p/CCNE1	Ghosh et al. (2017)
EOC	downregulation	tumor growth↓	colony formation↓, apoptosis↑, paclitaxel sensitivity↑, migration↓, invasion↓, chemoresistance↓	miR-874-3p, miR-874-5p/SIK2	Xia et al. (2018)
CC	downregulation	tumor growth↓	proliferation↓, migration↓, invasion↓		Liu et al. (2021a)
	downregulation	tumor growth↓	proliferation↓, apoptosis↑, migration↓, invasion↓	miR-874/ETS1	Liao et al. (2018)
PCa	downregulation				Pashaei et al. (2017)
TNBC	downregulation	tumor growth↓	proliferation↓, migration↓, invasion↓, EMT↓	lncRNA DANCR/miRNA-874-3p/SOX2	Wu et al. (2020)
BC	downregulation	tumor growth↓			Zhang et al. (2017)
	downregulation	tumor growth↓	proliferation↓, apoptosis↑, cell circle↓	miR-874/CDK9	Wang et al. (2014)
HNSCC	downregulation	tumor growth↓	proliferation↓, cell cycle↓, apoptosis↑	miR-874-3p/HDAC1	Nohata et al. (2013)
MSCC	downregulation	tumor growth↓	proliferation↓, invasion↓	miR-874-3p/PPP1CA	Nohata et al. (2011)
RB	downregulation	tumor growth↓	proliferation↓, migration↓, invasion↓, apoptosis↑	miR-874-3p/MTDH	Zhang et al. (2018a)
	downregulation	tumor growth↓	proliferation↓, migration↓, invasion↓, EMT↓	lncRNA TP73-AS1/miRNA-874-3p/TFAP2B/Wnt/β-catenin	Wang et al. (2020a)
RMS	downregulation	tumor growth↓	migration↓, invasion↓, apoptosis↑	miR-874-3p/GEFT	Shang et al. (2019)
Glioma	downregulation	tumor growth↓	proliferation↓, migration ↓, invasion↓		Li et al. (2021a)
Cholangiocarcinoma	downregulation	tumor growth↓	migration↓, invasion↓, EMT↓	miR-874-3p/CCNE1/NF-κB signailing pathway	Pan et al. (2021)
MM	downregulation	tumor growth↓	viability↓, proliferation↓, autophagy↓, apoptosis↑	circ_0003489/miR-874-3p/HDAC1	Tian et al. (2021)
PTC	downregulation	tumor growth↓	proliferation↓, migration↓, invasion↓, apoptosis↑, EMT↓	miR-874-3p/FAM84A/Wnt/β-catenin	Ding et al. (2021)
Endometrial cancer	downregulation	tumor growth↓	cell cycle↓		Witek et al. (2021)
AML	downregulation	tumor growth↓	autophagy↑	lncRNA DANCR/miR-874-3p/ATG16L1	Zhang et al. (2021a)

HCC, Hepatocellular carcinoma; PDAC, Pancreatic ductal adenocarcinoma; NSCLC, Non-small cell lung cancer; OS, Osteosarcoma; EOC, Epithelial ovarian cancer; CC, Cervical cancer; PCa, Prostate cancer; TNBC, Triple negative breast cancer; BC, Breast cancer; CRC, Colorectal cancer; ESCC, Esophageal squamous cell Squamous cell carcinoma; MSCC, Maxillary sinus squamous cell carcinoma; RB, Retinoblastoma; GC, Gastric cancer; RMS, Rhabdomyosarcoma; PTC, Papillary thyroid carcinoma; MM, myeloma; AML, acute myeloid leukemia; HNSCC, head and neck squamous cell carcinoma; CDF, Novel difluorobenzylidene analogue of curcumin; TSN, Tanshinone IIA; EMT, epithelial-mesenchymal transition.

HNSCC is the sixth most common cancer in the world (Nohata et al., 2013). The expression of miR-874-3p is reduced in HNSCC, and miR-874-3p can be upregulated after 5-Aza-dC treatment, indicating that the downregulation of miR-874-3p in HNSCC may be due to the methylation of its upstream CpG island (Nohata et al., 2013). Histone deacetylase 1 (HDAC1) belongs to the HDAC family. HDAC removes acetyl groups from histones and other nuclear proteins that induce chromatin condensation and transcriptional inhibition. HDAC plays an important role in the abnormal epigenetic changes associated with human cancer (Witt et al., 2009). In HNSCC, miR-874-3p can directly target HDAC1, significantly inhibit cell proliferation, and induce cell cycle arrest and apoptosis (Nohata et al., 2013).

Also, miR-874 plays an important role in non-cancer diseases. Low expression of miR-874 is associated with the risk of ischemic stroke (IS) (Jiang et al., 2019; Xie et al., 2020; Zhang et al., 2021b), ischemic heart disease (IHD) (Chen et al., 2019), cardiomyocyte necrosis (Wang et al., 2013), diabetic erectile dysfunction (DMED) (Huo et al., 2020), diabetic nephropathy (DN) (Yao et al., 2018), sepsis (Fang et al., 2018), osteoporosis (Lin et al., 2018), Parkinson’s disease (PD) (Zhao et al., 2020), acute myocardial infarction (AMI) (Yan et al., 2017), intestinal barrier dysfunction (Su et al., 2016), periodontal tissue repair and regeneration (Huang et al., 2020b), idiopathic short stature (ISS) (Liu et al., 2021b), intervertebral disc degeneration (IDD) (Song et al., 2021a), pneumonia (Yang et al., 2021), and human periodontal ligament fibroblasts (Song et al., 2021b) (Table 2).

**TABLE 2:** The role of miR-874 in human non-cancer diseases.

Non-cancer Type	The expression of miR-874	Effect *in vivo*	Effect *in vitro*	Regulatory mechanism	References
Ischemic stroke	downregulation	cerebral I/R injury↓	proliferation↑, apoptosis↓	miR-874-3p/BMF, BCL2L13	Jiang et al. (2019)
	downregulation		apoptosis↑	MIAT/miR-874-3p/IL1B	Zhang et al. (2021b)
	downregulation	angiogenesis↑, inflammatory factor release↓	apoptosis↓	miR-874-3p/CXCL12/Wnt/β-catenin	Xie et al. (2020)
Ischemic heart disease	downregulation	cardiac function↓	apoptosis↑	miR-874-3p/STAT3	Chen et al. (2019)
Cardiomyocyte necrosis	downregulation		apoptosis↑, necrosis↑	FOXO3/miR-874/CASP8	Wang et al. (2013)
Diabetic nephropathy	downregulation	inflammatory cytokines expression↓	proliferation↑, apoptosis↓	miR-874-3p/TLR4	Yao et al. (2018)
Diabetic erectile dysfunction	downregulation	erectile dysfunction↓	apoptosis↓	miR-874-3p/NUPR1/DDIT3	Huo et al. (2020)
Sepsis	downregulation	sepsis↓		lncRNA H19/miR-874/AQP1	Fang et al. (2018)
Periodontal tissue repair and regeneration				lncRNA FER1L4/miR-874-3p/VEGFA	Huang et al. (2020b)
Osteoporosis	downregulation		proliferation↑, apoptosis↓, osteoblasts in S phase↑, ALP activity↑, calcium nodules↑	miR-874-3p/SUFU/Hedgehog pathway	Lin et al. (2018)
Parkinson’s disease	downregulation		MPP + -induced neuronal injury↓	lncRNA HOTAIR/miR-874-5p/ATG10	Zhao et al. (2020)
Bone porosis	upregulation	osteoblast differentiation↑, mineralization↑		miR-874-3p/HDAC1/RUNX2	Kushwaha et al. (2016)
Acute myocardial infarction	downregulation		apoptosis↑	miR-874-3p/GZMB	Yan et al. (2017)
Intestinal barrier dysfunction	downregulation			lncRNA H19/miR-874-3p/AQP3	Su et al. (2016)
Intervertebral disc degeneration	downregulation			miR-874-3p/MMP2/MMP3	Song et al. (2021a)
	upregulation		proliferation↓, apoptosis↑	circ_0040039/miR-874-3p↑/ESR1	Li et al. (2021b)
Polycystic ovary syndrome and granulosa cell apoptosis	upregulation		apoptosis↑	miR-874-3p/HDAC1/p53 axis	Wei et al. (2021)
Idiopathic short stature	downregulation		proliferation↓, cell circle↓	circANAPC2/miR-874-3p/Smad3	Liu et al. (2021b)
Human periodontal ligament fibroblasts	downregulation		differentiation↑	miR-874-3p/WNT3A, WNT/β-catenin	Song et al. (2021b)
Pneumonia	downregulation		apoptosis↓	miR-874-3p/EGR3/NF-κB	Yang et al. (2021)

I/R, ischaemia/reperfusion; ALP, Alkaline phosphatase.

Highly expressed miR-874-3p is associated with the risk of bone porous (Kushwaha et al., 2016) and polycystic ovary syndrome (PCOS) (Wei et al., 2021), IDD (Li et al., 2021b). miR-874-3p in maternal osteoblasts increased 4–6 times during the child’s weaning period. Increasing the expression of miR-874-3p could enhance bone formation and restore the mother’s bone quality after pregnancy and lactation (Kushwaha et al., 2016). In granulosa cells, testosterone promotes p53 acetylation and expression by upregulating the expression of miR-874-3p and induces granulosa cell apoptosis (Wei et al., 2021), thereby promoting the occurrence and development of PCOS (Wei et al., 2021) (Table 2).

However, the results of the association between miR-874-3p expression and IDD are divergent. The expression level of miR-874-3p in the NP tissues of IDD patients was significantly reduced, thereby upregulating the expression of MMP2 and MMP3, eventually leading to the occurrence of IDD (Song et al., 2021a). In nucleus pulposus cells (NPCs), circ_0040039 can increase the stability of miR-874-3p and upregulate the miR-874-3p/ESR1 pathway to aggravate IDD (Li et al., 2021b). The different effects of miR-874-3p in IDD may be related to the tested sample types. It is worth noting that the sample size of IDD-related studies is small, and there is a lack of follow-up experiments to further explore the *in vivo* function of miR-874-3p. In the future, more samples and *in vivo* experiments are needed to confirm the mechanism of miR-874-3p in IDD.

## The Effect of miR-874 on Prognosis and Chemoresistance

In patients of HCC, OS, or RMS, decreased expression of miR-874 is associated with tumor size, vascular infiltration, lymph node metastasis, tumor-node-metastasis (TNM) staging, clinical staging, and tumor differentiation (Dong et al., 2016; Zhang et al., 2018b; Shang et al., 2019). Subsequent cell function experiments revealed the tumor suppressor effects of miR-874, including inhibition of proliferation, invasion, metastasis, and promotion of apoptosis (Diao et al., 2018) (Table 3).

**TABLE 3:** The contribution of miR-874 to the treatment, clinicopathological characteristics, and prognosis in cancer.

Cancer Type	Sample size	Expression pattern of miR-874	Radiosensitivity/Chemoresistance	Clinicopathological characteristics/Prognostic value	References
GC	50 paired tissues	downregulation	DDP, VCR, 5-FU	prognostic factor of OS	Huang et al. (2018)
CRC	32 paired tissues	downregulation	5-FU	prognostic factor of OS correlated with lymph node metastasis and TNM stage	Han et al. (2016)
	20 paired tissues	downregulation	5-FU		Que et al. (2017)
HCC	120 paired tissues	downregulation		prognostic factor of OS and RFS correlated with tumour size, vascular invasion, TNM stage, tumour differentiation and inferior patient outcomes	Zhang et al. (2018b)
ESCC	121 paired tissues	downregulation		independent prognostic factor of OS	Yuan et al. (2018)
NSCLC	32 paired tissues	downregulation	Radiosensitivity, chemoresistance		Bu et al. (2020)
	49 paired tissues	downregulation		prognostic factor of OS	Li et al. (2020)
PDAC	29 paired tissues	downregulation		correlated with TNM stage, tumor size, and lymph node metastasis	Diao et al. (2018)
OS	40 paired tissues	downregulation		correlated with TNM stage, tumor size, and lymph node metastasis	Dong et al. (2016)
EOC	20 paired tissues	downregulation	chemoresistance		Xia et al. (2018)
CC	49 paired tissues	downregulation		prognostic factor of OS correlated with tumour differentiation and lymph node metastasis	Liao et al. (2018)
BC	26 paired tissues	downregulation		correlated with pathological differentiation and tumor size	Wang et al. (2014)
	47 paired tissues	downregulation		prognostic factor of OS correlated with pathological differentiation, TNM staging and lymph node metastasis	Zhang et al. (2017)
RB	26 paired tissues	downregulation		correlated with tumor stage	Zhang et al. (2018a)
	50 paired tissues	downregulation		prognostic factor of OS correlated with different clinicopathological stage	Wang et al. (2020a)
RMS	10 paired tissues	downregulation		correlated with the advanced clinical stage, lymph node metastasis, and distant metastasis of RMS	Shang et al. (2019)
AML	HL60, U937, and KG1a	downregulation	Ara-C		Zhang et al. (2021a)
MM	MM1.R	downregulation	BTZ		Tian et al. (2021)
Glioma	105 paired tissues	downregulation		independent prognostic factor for OS correlated with tumor size, KPS score, and TNM stage	Li et al. (2021a)

GC, Gastric cancer; CRC, Colorectal cancer; HCC, Hepatocellular carcinoma; ESCC, Esophageal squamous cell Squamous cell carcinoma; NSCLC, Non-small cell lung cancer; PDAC, Pancreatic ductal adenocarcinoma; OS, Osteosarcoma; EOC, Epithelial ovarian cancer; CC, Cervical cancer; r BC, Breast cancer; RB, Retinoblastoma; RMS, Rhabdomyosarcoma; MM, myeloma; AML, acute myeloid leukemia; TNM, Tumor node metastasis; OS, Overall survival rate; RFS, Recurrence free survival.

As shown in Table 3, compared with cancer patients with high miR-874 expression, patients with low miR-874 expression have a significantly worse prognosis. These cancers include GC (Huang et al., 2018), CRC (Han et al., 2016), HCC (Zhang et al., 2018b), ESCC (Yuan et al., 2018), NSCLC (Li et al., 2020), CC (Liao et al., 2018), BC (Zhang et al., 2017), RB (Wang et al., 2020a), and glioma (45).

In GC, CRC, NSCLC, and EOC, miR-874 can reduce the drug resistance of cancer cells (Huang et al., 2018; Han et al., 2016; Que et al., 2017; Bu et al., 2020; Xia et al., 2018). Among them, the upregulation of miR-874 expression in CRC cells can increase the sensitivity to 5-FU (Han et al., 2016; Que et al., 2017). The overexpression of miR-874 significantly enhanced the sensitivity of GC cells to DDP, VCR, and 5-FU (Huang et al., 2018). The MIR210HG/miR-874/STAT3 axis plays a carcinogenic regulatory role in the radiosensitivity and drug resistance of NSCLC (Bu et al., 2020). miR-874-3p and miR-874-5p can enhance the chemical sensitivity of EOC cells (Xia et al., 2018).

Cancer cells use autophagy to provide energy and develop resistance to anti-cancer drugs; therefore, inhibiting autophagy may promote cancer cell death and help overcome drug resistance (Levy et al., 2017). Autophagy involved in miR-874-3p is an important mechanism for regulating chemotherapy resistance in AML (Zhang et al., 2021a). In AML, the DANCR/miR-874-3p/ATG16L1 axis can promote autophagy, thereby enhancing the resistance of human AML cells to Ara-C (Zhang et al., 2021a). In addition, knocking out circ_0003489 can upregulate miR-874-3p and inhibit HDAC1, thereby prompting MM cells to switch from autophagy to apoptosis, and reducing the growth of MM cells (Tian et al., 2021).

The above findings indicate that miR-874 can be developed as a new diagnostic and prognositc biomarker for patients with the above cancer types and suggest a potential value of miR-874 in cancer drug resistance.

## The ceRNA Network Centered on miR-874

**FIGURE 2:**
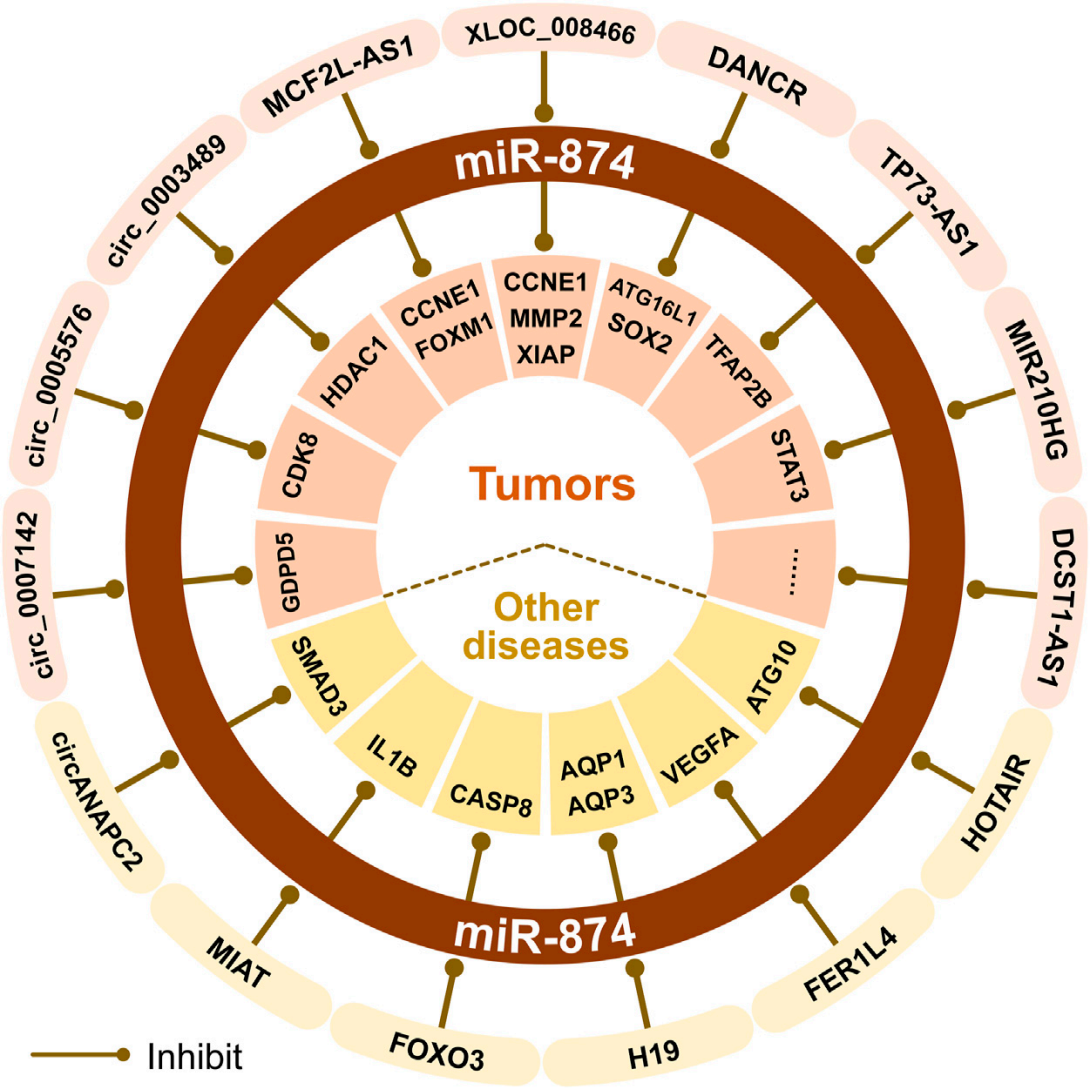
The ceRNA mechanisms of miR-874 in cancer and other non-cancer diseases.

Non-coding RNA can regulate gene expression, thereby affecting cell proliferation, survival, and migration, and is related to genome stability and malignant transformation of inflammatory cells (Zhang et al., 2021a). There are interactions between non-coding RNAs. For example, lncRNAs and circRNAs can be used as ceRNAs to sponge miRNAs (Zhang et al., 2021a). The ceRNA network centered on miR-874 is involved with at least 10 lncRNAs or 12 circRNAs. The dysfunction of miR-874 is closely related to the occurrence and development of tumors and other diseases (Figure 2).

In TNBC, lncRNA DANCR acts as a ceRNA for miR-874-3p, thereby regulating the derepression of SOX2 and promoting the EMT in TNBC (Wu et al., 2020). In CRC, the MCF2LAS1/miR-874-3p/FOXM1 axis (Liao et al., 2018) and MCF2LAS1/miR-874-3p/CCNE1 axis (Huang et al., 2020a) can promote cancer cell apoptosis, inhibit cancer cell proliferation, invasion, migration and EMT process. Also, the circ_0005576/miR-874/CDK8 axis can promote the malignant progression of CRC (Yu et al., 2020). In CRC cells, circ_0007142 can regulate the level of GDPD5 by sponging miR-874-3p (12). Knock-down of circ_0007142 can induce ferroptosis through the circ_0007142/miR-874-3p/GDPD5 axis, thereby increasing the effectiveness of chemotherapy or radiotherapy and inhibiting the malignant progression of CRC (Wang et al., 2021). In RB, the expression of lncRNA TP73-AS1 is upregulated, and the downregulated TP73-AS1/miR-874-3p/TFAP2B axis can inhibit the Wnt/β-catenin signaling pathway, thereby inhibiting tumor progression (Wang et al., 2020a). In NSCLC, the miR210HG/miR-874-3p/STAT3 axis plays a role in the progression of NSCLC cells (Bu et al., 2020). Besides, XLOC_008466, as the ceRNA of miR-874-3p, can increase the expression of MMP2 and XIAP and affect NSCLC cell proliferation, apoptosis, and invasion (Yang et al., 2017; Bu et al., 2020). In OS, the downregulated lncRNA XLOC_008466/miR-874-3p/CCNE1 axis can also inhibit tumor growth (Ghosh et al., 2017). The expression of lncRNA DCST1-AS1 increases in cervical cancer tissues and cells, and inhibition of DCST1-AS1 can increase the expression of miR-874-3p, thereby inhibiting the proliferation, migration and invasion of cervical cancer cells (Liu et al., 2021a). In PTC, miR-874-3p can inhibit FAM84A and exert carcinogenic effects through the Wnt/β-catenin signal transduction (Ding et al., 2021). LncRNA DANCR is a promising tumor-related lncRNA that can enhance cancer cell proliferation, stemness, invasion, and metastasis (Zhang et al., 2021a). In AML, DANCR regulates autophagy by promoting the miR-874-3p/ATG16L1 axis, thereby reducing Ara-C resistance in human AML cells (Zhang et al., 2021a). BTZ is a first-class proteasome inhibitor approved by the FDA for the treatment of newly diagnosed and relapsed MM patients. In MM, the circ_0003489/miR-874-3p/HDAC1 axis plays a crucial role in controlling the balance of autophagy and apoptosis in MM cells. Downregulation of circ_0003489 can increase the inhibition of miR-874-3p on HDAC1, and improve the efficacy of BTZ in the treatment of MM (Tian et al., 2021).

Besides, we found that the ceRNA network of miR-874 also plays an important role in non-cancer diseases. The H19/miR-874/AQP1 axis can help restore inflammatory response to lipopolysaccharide (LPS) and inflammation associated with sepsis-induced myocardial dysfunction (Fang et al., 2018). Also, the H19/miR-874/AQP3 axis plays an important role in maintaining the intestinal barrier function (Su et al., 2016). During the continuous osteogenic differentiation of periodontal ligament stromal cells (PDLSCs), the FER1L4/miR-874-3p/VEGFA axis can positively regulate the osteogenic differentiation of PDLSCs (Huang et al., 2020b). In PD, the HOTAIR/miR-874-5p/ATG10 axis can promote MPP^+^-induced neuronal damage (Zhao et al., 2020). miR-874-3p can reduce the levels of TNF-α, IL-1, IL-6, and IL-8, increase the level of IL-10, reduce neuronal apoptosis, and significantly inhibit brain inflammation in the IS model mice. LncRNA MIAT can sponge miR-874-3p to increase the risk of IS (Zhang et al., 2021b). In addition, circANAPC2 is upregulated in ISS patients, and it can inhibit the proliferation, hypertrophy, and endochondral ossification of chondrocytes through the circANAPC2/miR-874-3p/SMAD3 axis *in vitro* (Liu et al., 2021b).

It is worth noting that circRNA-mediated regulation of miRNA expression consists of two modes (Piwecka et al., 2017). One is the classical sponge mechanism, in which circRNA inhibits or does not affect miRNA expression. The other is a stabilization mechanism in which circRNAs increase the expression of miRNAs. In IDD, circ_0040039 can stabilize miR-874-3p and inhibit the expression level of ESR1, thereby promoting the apoptosis of the NPCs and inhibiting the growth of NPCs (Li et al., 2021b).

## The miR-874 Related Signaling Pathways

**FIGURE 3:**
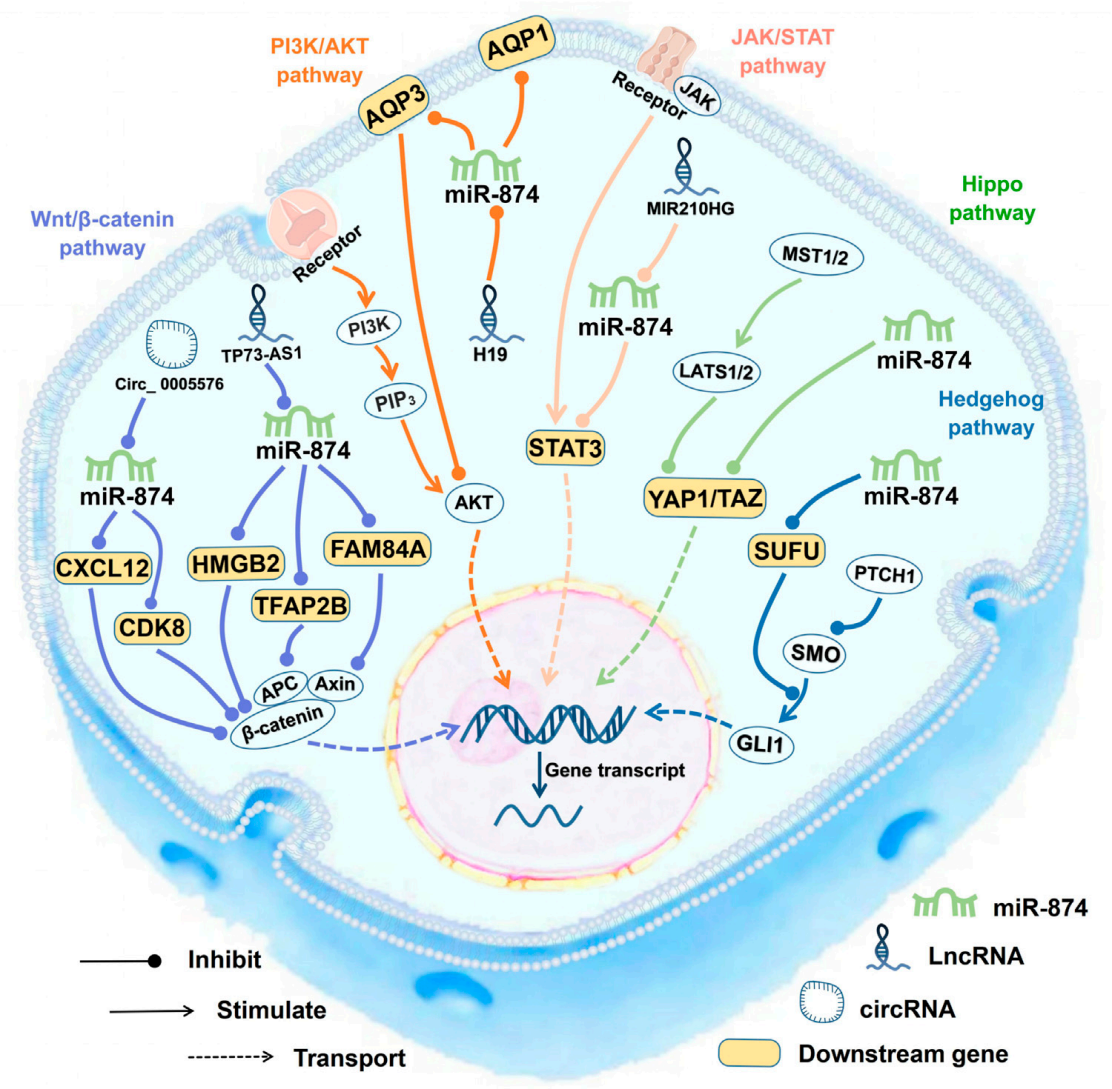
miR-874 regulate many biological processes within the cell. The target genes of miR-874 are involved in at least five classical signaling pathways, including the Wnt/β-catenin signaling pathway, P13/AKT signaling pathway, Hippo signaling pathway, JAK/STAT signaling pathway, and Hedgehog signaling pathway. EMT, epithelial-mesenchymal transition.

The target genes of miR-874 are involved in at least five classical signaling pathways, including the Wnt/β-catenin pathway [TFAP2B (Wang et al., 2020a), CDK8 (Yu et al., 2020), HMGB2 (Yuan et al., 2020), CXCL12 (Xie et al., 2020), and FAM84A (Ding et al., 2021)], the Hippo pathway [YAP1 and TAZ (Que et al., 2017)], the PI3K/AKT pathway [AQP3 (Li et al., 2020)], the JAK/STAT pathway [STAT3 (Zhang et al., 2015; Li et al., 2020; Zhao and Dong, 2016; Bu et al., 2020; Yuan et al., 2018; Shang et al., 2019)] and the Hedgehog signaling pathway [SUFU (Lin et al., 2018)] (Figure 3).

### The Wnt/β-Catenin Signaling Pathway

The close relationship between miR-874 and Wnt/β-catenin signaling pathway is of great significance not only for tumor diseases but also for IS (Figure 3). Wnt/β-catenin signaling pathway plays a key role in regulating cell growth, cell development, and normal stem cell differentiation. Constitutive activation of the Wnt/β-catenin signaling pathway has been found in many human cancers (Yao et al., 2011). In RB tissues and cells, the TP73-AS1/miR-874-3p/TFAP2B axis can activate the Wnt/β-catenin signaling pathway and enhance the expression of downstream tumor-related factors TCF4, BCL2, CCND1, and MYC (Wang et al., 2020a).

Abnormal activation of the Wnt/β-catenin signaling pathway occurs in almost all CRC (Wang et al., 2020a). In CRC, the circ_0005576/miR-874-3p/CDK8 axis can cause the abnormal activation of the Wnt/β-catenin signaling pathway and the proliferation of CRC cells (Yu et al., 2020). In GC cell lines the downregulation of the miR-874-3p/HMGB2 axis can upregulate the expression of β-catenin, CCND1, and MYC, which shows that the abnormal activation Wnt/β-catenin signaling pathway may be regulated by the miR-874-3p/HMGB2 axis in GC (Yuan et al., 2020).

The expression of serum CXCL12 in patients with IS was higher than that in healthy controls. CXCL12 can act as a ligand for CXC motif chemokine receptor 4 (CXCR4) and is a downstream target gene of miR-874-3p (Xie et al., 2020). In mice with IS, the Wnt/β-catenin signaling pathway is inhibited, and downregulation of CXCL12 can activate the Wnt/β-catenin signaling pathway, thereby promoting angiogenesis and inhibiting the brain tissue apoptosis in mice with IS (Xie et al., 2020). This suggests that the miR-874-3p/CXCL12 axis can activate the Wnt/β-catenin signaling pathway, which provides a new hint for the treatment of IS (Xie et al., 2020).

In PTC, FAM84A can activate EMT and the Wnt/β-catenin signaling pathway, thereby inducing tumorigenesis of thyroid cancer (Ding et al., 2021). miR-874-3p can target the 3′UTR of FAM84A, thereby reducing the expression of FAM84A. Attenuation of miR-874-3p/FAM84A/Wnt/β-catenin axis can inhibit PTC tumor progression (Ding et al., 2021).

In addition, miR-874-3p/WNT/β-catenin axis can inhibit the osteogenic differentiation of hPDLF. During osteogenic differentiation of hPDLF, the downregulation of miR-874-3p corresponds to the increase in WNT3A expression, while overexpression of miR-874-3p can inhibit WNT3A expression, thereby upregulating the expression of the β-catenin protein (Song et al., 2021b).

### The Hippo Signaling Pathway

The Hippo signaling pathway can regulate cell growth, differentiation, aging, contact inhibition, and other biological processes, and plays an important role in maintaining cell growth and maintaining the stability of apoptosis balance (Que et al., 2017). YAP1 and TAZ are downstream transcriptional effectors of the Hippo signaling pathway, which can promote cell growth, invasion, and migration (Que et al., 2017). In CRC cells, the ectopic expression of miR-874-3p can inhibit the expression of YAP1 and TAZ, and by downregulating the expression of BCL2 and BCL2L1, increasing the activity of CASP9 and CASP3, thereby promoting 5-FU-induced apoptosis (Que et al., 2017). Downregulation of miR-874-3p can inactivate the Hippo signaling pathway, thereby increasing the resistance of cells to 5-FU chemotherapy (Que et al., 2017) (Figure3).

### The PI3K/AKT Signaling Pathway

The PI3K/AKT signaling pathway is downstream of many growth factor receptors. It promotes the proliferation and malignant transformation of tumor cells and inhibits tumor cell apoptosis through the phosphorylation of PI3K and AKT proteins (Lu et al., 2019). Downregulation of miR-874 in NSCLC tissues and cell lines can increase the expression of its target gene AQP3, promote p-PI3K and p-AKT phosphorylation, and activate the PI3K/AKT signaling pathway (Wang et al., 2020b). The above implies that miR-874 deactivates the PI3K/AKT signaling pathway by targeting AQP3 and exerts its tumor suppressor effect (Wang et al., 2020b) (Figure 3).

### The JAK/STAT Signaling Pathway

The JAK/STAT signaling pathway includes a family of receptor-associated cytoplasmic tyrosine kinases (JAKs) that phosphorylate tyrosine residues in STAT homologs (Wang et al., 2019). The JAK/STAT signaling pathway plays an inhibitory role in various physiological processes, such as cell development and differentiation (Wang et al., 2019).

miR-874-3p can inhibit the JAK/STAT signaling pathway by inhibiting STAT3 (Figure 3). As an anti-apoptotic factor, STAT3 plays an important role in the regulation of gene expression and mitochondrial electron transport during cellular stress (Wang et al., 2019). miR-874-3p can inhibit STAT3 in several cancers, including GC (Zhang et al., 2015), CRC (Zhao and Dong, 2016), NSCLC (Bu et al., 2020), and ESCC (Yuan et al., 2018). In gastric cancer, constitutive STAT3 activation promotes VEGF-A expression and stimulates tumor angiogenesis. miR-874 can bind to the 3′-UTR of STAT3 and downregulate STAT3 expression, thereby inhibiting angiogenesis (Zhang et al., 2015). In CRC, miR-874 inhibits STAT3 expression by targeting its mRNA 3′UTR, thereby inhibiting cell growth and inducing apoptosis (Zhao and Dong, 2016). In NSCLC cells, miR210HG can downregulate the expression of miR-874, thereby promoting the expression of STAT3 (Bu et al., 2020). In ESCC, the overexpression of miR-874 can inhibit tumor development by targeting STAT3. Besides, in IHD, inhibiting miR-874-3p can activate the JAK2/STAT3 signaling pathway, thereby inhibiting the expression of BAX, upregulating BCL2, reducing cardiomyocyte apoptosis, and ultimately reducing the risk of ischaemia/reperfusion (I/R) damage in mice (Chen et al., 2019).

### The Hedgehog Signaling Pathway

The Hedgehog signaling pathway is conservative and it is involved in the proliferation and differentiation of a variety of cells (Lin et al., 2018). SUFU is a negative regulator of the Hedgehog signaling pathway in vertebrates. SUFU can inhibit the GLI transcription factor and induce skeletal dysplasia, osteoarthritis, or chondroma (Lin et al., 2018). By inhibiting SUFU and activating the Hedgehog signaling pathway, miR-874 can promote osteoblast proliferation, increase alkaline phosphatase activity and calcium nodules, and inhibit osteoblast apoptosis (Lin et al., 2018).

## Summary

miR-874 is downregulated in many cancers and non-cancer diseases, suggesting that it plays a key role in the physiological and pathological processes of human disease. miR-874 plays an important role in the progression of malignant tumors by regulating a complex ceRNA network. The ceRNA network centered on miR-874 includes at least 10 ncRNAs and 12 protein-coding genes. miR-874 has also been shown to participate in at least 4 important signaling pathways, including the Hippo signaling pathway, Wnt/β-catenin signaling pathway, JAK/STAT signaling pathway, and Hedgehog signaling pathway.

It is worth noting that in the relevant research of IDD, the expression of miR-874-3p is inconsistent. This may be related to the cell state and type, and these differences need to be further verified in large-scale experiments. In IDD, circ_0040039 can enhance miR-874-3p through a stabilization mechanism. In the future, further exploration of miR-874-related stabilization mechanisms will help to understand the ceRNA network of miR-874 and the clinical effectiveness of targeting miR-874.

The abnormal expression of miR-874 is closely related to the clinicopathological characteristics of 15 cancers. Therefore, miR-874 can be used as a potential biomarker for the early prediction of cancer. In addition, in AML and MM, miR-874 participates in the regulation of autophagy-related functions and affects drug resistance of cells, which provides new ideas for overcoming drug resistance. However, the current research of miR-874 is focused on the exploration of the mechanism of its upstream and downstream genes. The potential clinical application of miR-874 in cancer prognosis and chemotherapy resistance is still lacking.

In existing studies, miR-874 is downregulated in all cancers studied and is related to the clinicopathological characteristics of cancer. Therefore, miR-874 is promising as a potential biomarker for the early prediction of cancer. In addition, in recent years, more and more non-cancer diseases have also recognized the evidence related to miR-874, but the specific regulatory mechanism of miR-874 in non-cancer diseases remains to be revealed. Future work is necessary to explore the mechanism of miR-874-related ceRNA network in cancer and non-cancer disease.

## References

[B1] Ahmad A. Sayed A. Ginnebaugh K. R. Sharma V. Suri A. Saraph A. (2015). Molecular Docking and Inhibition of Matrix Metalloproteinase-2 by Novel Difluorinatedbenzylidene Curcumin Analog. Am. J. Transl Res. 7 (2), 298–308. 25901198 PMC4399093

[B2] Bu L. Zhang L. Tian M. Zheng Z. Tang H. Yang Q. (2020). LncRNA MIR210HG Facilitates Non-small Cell Lung Cancer Progression through Directly Regulation of miR-874/STAT3 Axis. Dose Response 18 (3), 1559325820918052. 10.1177/1559325820918052 32699535 PMC7357071

[B3] Chen P. J. Shang A. Q. Yang J. P. Wang W. W. (2019). microRNA-874 Inhibition Targeting STAT3 Protects the Heart from Ischemia-Reperfusion Injury by Attenuating Cardiomyocyte Apoptosis in a Mouse Model. J. Cel Physiol 234 (5), 6182–6193. 10.1002/jcp.27398 30370578

[B4] Diao J. Su X. Cao L. Yang Y. Liu Y. (2018). MicroRNA874 Inhibits Proliferation and Invasion of Pancreatic Ductal Adenocarcinoma Cells by Directly Targeting Paired Box 6. Mol. Med. Rep. 18 (1), 1188–1196. 10.3892/mmr.2018.9069 29845293

[B5] Ding Y. Wu L. Zhuang X. Cai J. Tong H. Si Y. (2021). The Direct miR-874-3p-Target FAM84A Promotes Tumor Development in Papillary Thyroid Cancer. Mol. Oncol. 15 (5), 1597–1614. 10.1002/1878-0261.12941 33751775 PMC8096794

[B6] Dong D. Gong Y. Zhang D. Bao H. Gu G. (2016). miR-874 Suppresses the Proliferation and Metastasis of Osteosarcoma by Targeting E2F3. Tumour Biol. 37 (5), 6447–6455. 10.1007/s13277-015-4527-3 26631042

[B7] Fang Y. Hu J. Wang Z. Zong H. Zhang L. Zhang R. (2018). LncRNA H19 Functions as an Aquaporin 1 Competitive Endogenous RNA to Regulate microRNA-874 Expression in LPS Sepsis. Biomed. Pharmacother. 105, 1183–1191. 10.1016/j.biopha.2018.06.007 30021355

[B8] Ghosh T. Varshney A. Kumar P. Kaur M. Kumar V. Shekhar R. (2017). MicroRNA-874-mediated Inhibition of the Major G1/S Phase Cyclin, CCNE1, Is Lost in Osteosarcomas. J. Biol. Chem. 292 (52), 21264–21281. 10.1074/jbc.M117.808287 29109143 PMC5766939

[B9] Han J. Liu Z. Wang N. Pan W. (2016). MicroRNA-874 Inhibits Growth, Induces Apoptosis and Reverses Chemoresistance in Colorectal Cancer by Targeting X-Linked Inhibitor of Apoptosis Protein. Oncol. Rep. 36 (1), 542–550. 10.3892/or.2016.4810 27221209

[B10] Huang F. K. Zheng C. Y. Huang L. K. Lin C. Q. Zhou J. F. Wang J. X. (2020a). Long Non-coding RNA MCF2L-AS1 Promotes the Aggressiveness of Colorectal Cancer by Sponging miR-874-3p and Thereby Up-Regulating CCNE1. J. Gene Med. 36, e3285. 10.1002/jgm.3285 33037865

[B11] Huang H. Tang J. Zhang L. Bu Y. Zhang X. (2018). miR-874 Regulates Multiple-Drug Resistance in Gastric Cancer by Targeting ATG16L1. Int. J. Oncol. 53 (6), 2769–2779. 10.3892/ijo.2018.4593 30320370

[B12] Huang Y. Han Y. Guo R. Liu H. Li X. Jia L. (2020b). Long Non-coding RNA FER1L4 Promotes Osteogenic Differentiation of Human Periodontal Ligament Stromal Cells via miR-874-3p and Vascular Endothelial Growth Factor A. Stem Cel Res Ther 11 (1), 5. 10.1186/s13287-019-1519-z PMC6942378 31900200

[B13] Huo W. Li H. Zhang Y. Li H. (2020). Epigenetic Silencing of microRNA-874-3p Implicates in Erectile Dysfunction in Diabetic Rats by Activating the Nupr1/Chop-Mediated Pathway. FASEB J. 34 (1), 1695–1709. 10.1096/fj.201902086R 31914690

[B14] Iorio M. V. Croce C. M. (2017). MicroRNA Dysregulation in Cancer: Diagnostics, Monitoring and Therapeutics. A Comprehensive Review. EMBO Mol. Med. 9 (6), 852. 10.15252/emmm.201707779 28572088 PMC5452025

[B15] Jiang B. Li Z. Zhang W. Wang H. Zhi X. Feng J. (2014). miR-874 Inhibits Cell Proliferation, Migration and Invasion through Targeting Aquaporin-3 in Gastric Cancer. J. Gastroenterol. 49 (6), 1011–1025. 10.1007/s00535-013-0851-9 23800944

[B16] Jiang D. Sun X. Wang S. Man H. (2019). Upregulation of miR-874-3p Decreases Cerebral Ischemia/reperfusion Injury by Directly Targeting BMF and BCL2L13. Biomed. Pharmacother. 117, 108941. 10.1016/j.biopha.2019.108941 31200256

[B17] Jiang T. Guan L. Y. Ye Y. S. Liu H. Y. Li R. (2017). MiR-874 Inhibits Metastasis and Epithelial-Mesenchymal Transition in Hepatocellular Carcinoma by Targeting SOX12. Am. J. Cancer Res. 7 (6), 1310–1321. 28670493 PMC5489780

[B18] Kesanakurti D. Maddirela D. R. Chittivelu S. Rao J. S. Chetty C. (2013). Suppression of Tumor Cell Invasiveness and *In Vivo* Tumor Growth by microRNA-874 in Non-small Cell Lung Cancer. Biochem. Biophys. Res. Commun. 434 (3), 627–633. 10.1016/j.bbrc.2013.03.132 23583374

[B19] Kushwaha P. Khedgikar V. Sharma D. Yuen T. Gautam J. Ahmad N. (2016). MicroRNA 874-3p Exerts Skeletal Anabolic Effects Epigenetically during Weaning by Suppressing Hdac1 Expression. J. Biol. Chem. 291 (8), 3959–3966. 10.1074/jbc.M115.687152 26663087 PMC4759174

[B20] Leong K. W. Cheng C. W. Wong C. M. Ng I. O. Kwong Y. L. Tse E. (2017). miR-874-3p Is Down-Regulated in Hepatocellular Carcinoma and Negatively Regulates PIN1 Expression. Oncotarget 8 (7), 11343–11355. 10.18632/oncotarget.14526 28076852 PMC5355269

[B21] Levy J. M. M. Towers C. G. Thorburn A. (2017). Targeting Autophagy in Cancer. Nat. Rev. Cancer 17 (9), 528–542. 10.1038/nrc.2017.53 28751651 PMC5975367

[B22] Li Y. Chen X. Xue W. Liang J. Wang L. (2021a). MiR-874 Inhibits Cell Proliferation, Migration, and Invasion of Glioma Cells and Correlates with Prognosis of Glioma Patients. Neuromolecular Med. 23 (2), 247–255. 10.1007/s12017-020-08608-0 32803522

[B23] Li Y. L. Wang X. M. Qiao G. D. Zhang S. Wang J. Cong Y. Z. (2020). Up-regulated Lnc-Lung Cancer Associated Transcript 1 Enhances Cell Migration and Invasion in Breast Cancer Progression. Biochem. Biophys. Res. Commun. 521 (2), 271–278. 10.1016/j.bbrc.2019.08.040 31635802

[B24] Li Y. Wang X. Xu H. Li G. Huo Z. Du L. (2021b). Circ_0040039 May Aggravate Intervertebral Disk Degeneration by Regulating the MiR-874-3p-ESR1 Pathway. Front. Genet. 12, 656759. 10.3389/fgene.2021.656759 34178027 PMC8226233

[B25] Liao H. Pan Y. Pan Y. Shen J. Qi Q. Zhong L. (2018). MicroRNA874 Is Downregulated in Cervical Cancer and Inhibits Cancer Progression by Directly Targeting ETS1. Oncol. Rep. 40 (4), 2389–2398. 10.3892/or.2018.6624 30106442

[B26] Lin J. C. Liu Z. G. Yu B. Zhang X. R. (2018). MicroRNA-874 Targeting SUFU Involves in Osteoblast Proliferation and Differentiation in Osteoporosis Rats through the Hedgehog Signaling Pathway. Biochem. Biophys. Res. Commun. 506 (1), 194–203. 10.1016/j.bbrc.2018.09.187 30342851

[B27] Liu B. Li F. Zhao H. P. Chen J. B. Li Y. P. Yu H. H. (2017). miR-874 Inhibits Metastasis-Relevant Traits via Targeting SH2B Adaptor Protein 1 (SH2B1) in Gastric Cancer. Int. J. Clin. Exp. Pathol. 10 (8), 8577–8584. 31966712 PMC6965372

[B28] Liu J. Zhang J. Hu Y. Zou H. Zhang X. Hu X. (2021a). Inhibition of lncRNA DCST1-AS1 Suppresses Proliferation, Migration and Invasion of Cervical Cancer Cells by Increasing miR-874-3p Expression. J. Gene Med. 23 (1), e3281. 10.1002/jgm.3281 33025624

[B29] Liu W. G. Zhuo L. Lu Y. Wang L. Ji Y. X. Guo Q. (2020). miR-874-3p Inhibits Cell Migration through Targeting RGS4 in Osteosarcoma. J. Gene Med. 22 (9), e3213. 10.1002/jgm.3213 32386256

[B30] Liu X. Du Z. Yi X. Sheng T. Yuan J. Jia J. (2021b). Circular RNA circANAPC2 Mediates the Impairment of Endochondral Ossification by miR-874-3p/SMAD3 Signalling Pathway in Idiopathic Short Stature. J. Cel Mol Med 25 (7), 3408–3426. 10.1111/jcmm.16419 PMC8034469 33713570

[B31] Lu T. X. Rothenberg M. E. (2018). MicroRNA. J. Allergy Clin. Immunol. 141 (4), 1202–1207. 10.1016/j.jaci.2017.08.034 29074454 PMC5889965

[B32] Lu Y. Li L. Wu G. Zhuo H. Liu G. Cai J. (2019). Effect of PI3K/Akt Signaling Pathway on PRAS40Thr246 Phosphorylation in Gastric Cancer Cells. Iran J. Public Health 48 (12), 2196–2204. 31993387 PMC6974862

[B33] Nohata N. Hanazawa T. Kikkawa N. Sakurai D. Fujimura L. Chiyomaru T. (2011). Tumour Suppressive microRNA-874 Regulates Novel Cancer Networks in Maxillary Sinus Squamous Cell Carcinoma. Br. J. Cancer 105 (6), 833–841. 10.1038/bjc.2011.311 21847129 PMC3171017

[B34] Nohata N. Hanazawa T. Kinoshita T. Inamine A. Kikkawa N. Itesako T. (2013). Tumour-suppressive microRNA-874 Contributes to Cell Proliferation through Targeting of Histone Deacetylase 1 in Head and Neck Squamous Cell Carcinoma. Br. J. Cancer 108 (8), 1648–1658. 10.1038/bjc.2013.122 23558898 PMC3668462

[B35] Pan X. Wang G. Wang B. (2021). Ectopic Expression of microRNA-874 Represses Epithelial Mesenchymal Transition through the NF-kappaB Pathway via CCNE1 in Cholangiocarcinoma. Cell Signal 82, 109927. 10.1016/j.cellsig.2021.109927 33476715

[B36] Pashaei E. Pashaei E. Ahmady M. Ozen M. Aydin N. (2017). Meta-analysis of miRNA Expression Profiles for Prostate Cancer Recurrence Following Radical Prostatectomy. PLoS One 12 (6), e0179543. 10.1371/journal.pone.0179543 28651018 PMC5484492

[B37] Piwecka M. Glazar P. Hernandez-Miranda L. R. Memczak S. Wolf S. A. Rybak-Wolf A. (2017). Loss of a Mammalian Circular RNA Locus Causes miRNA Deregulation and Affects Brain Function. Science 357 (6357). 10.1126/science.aam8526 28798046

[B38] Que K. Tong Y. Que G. Li L. Lin H. Huang S. (2017). Downregulation of miR-874-3p Promotes Chemotherapeutic Resistance in Colorectal Cancer via Inactivation of the Hippo Signaling Pathway. Oncol. Rep. 38 (6), 3376–3386. 10.3892/or.2017.6041 29039607 PMC5783584

[B39] Shang H. Liu Y. Li Z. Liu Q. Cui W. Zhang L. (2019). MicroRNA-874 Functions as a Tumor Suppressor in Rhabdomyosarcoma by Directly Targeting GEFT. Am. J. Cancer Res. 9 (4), 668–681. 31105995 PMC6511638

[B40] Song Q. Zhang F. Wang K. Chen Z. Li Q. Liu Z. (2021a). MiR-874-3p Plays a Protective Role in Intervertebral Disc Degeneration by Suppressing MMP2 and MMP3. Eur. J. Pharmacol. 895, 173891. 10.1016/j.ejphar.2021.173891 33482178

[B41] Song S. Yan Z. Wu W. (2021b). MiR-874-3p Inhibits Osteogenic Differentiation of Human Periodontal Ligament Fibroblasts through Regulating Wnt/beta-Catenin Pathway. J. Dent Sci. 16 (4), 1146–1153. 10.1016/j.jds.2021.02.006 34484582 PMC8403793

[B42] Song X. Song W. Wang Y. Wang J. Li Y. Qian X. (2016). MicroRNA-874 Functions as a Tumor Suppressor by Targeting Cancer/Testis Antigen HCA587/MAGE-C2. J. Cancer 7 (6), 656–663. 10.7150/jca.13674 27076846 PMC4829551

[B43] Su Z. Zhi X. Zhang Q. Yang L. Xu H. Xu Z. (2016). LncRNA H19 Functions as a Competing Endogenous RNA to Regulate AQP3 Expression by Sponging miR-874 in the Intestinal Barrier. FEBS Lett. 590 (9), 1354–1364. 10.1002/1873-3468.12171 27059301

[B44] Sun K. Lai E. C. (2013). Adult-specific Functions of Animal microRNAs. Nat. Rev. Genet. 14 (8), 535–548. 10.1038/nrg3471 23817310 PMC4136762

[B45] Sun Q. H. Yin Z. X. Li Z. Tian S. B. Wang H. C. Zhang F. X. (2020). miR-874 Inhibits Gastric Cancer Cell Proliferation by Targeting SPAG9. BMC Cancer 20 (1), 522. 10.1186/s12885-020-06994-z 32503577 PMC7275545

[B46] Tang W. Wang W. Zhao Y. Zhao Z. (2018). MicroRNA-874 Inhibits Cell Proliferation and Invasion by Targeting Cyclin-dependent Kinase 9 in Osteosarcoma. Oncol. Lett. 15 (5), 7649–7654. 10.3892/ol.2018.8294 29725464 PMC5920401

[B47] Tian F. Q. Chen Z. R. Zhu W. Tang M. Q. Li J. H. Zhang X. C. (2021). Inhibition of Hsa_circ_0003489 Shifts Balance from Autophagy to Apoptosis and Sensitizes Multiple Myeloma Cells to Bortezomib via miR-874-3p/HDAC1 axis. J. Gene Med. 23 (9), e3329. 10.1002/jgm.3329 33625798

[B48] Wang K. Liu F. Zhou L. Y. Ding S. L. Long B. Liu C. Y. (2013). miR-874 Regulates Myocardial Necrosis by Targeting Caspase-8. Cell Death Dis 4, e709. 10.1038/cddis.2013.233 23828572 PMC3730407

[B49] Wang L. Gao W. Hu F. Xu Z. Wang F. (2014). MicroRNA-874 Inhibits Cell Proliferation and Induces Apoptosis in Human Breast Cancer by Targeting CDK9. FEBS Lett. 588 (24), 4527–4535. 10.1016/j.febslet.2014.09.035 25281924

[B50] Wang L. Wang C. Wu T. Sun F. (2020a). Long Non-coding RNA TP73-AS1 Promotes TFAP2B-Mediated Proliferation, Metastasis and Invasion in Retinoblastoma via Decoying of miRNA-874-3p. J. Cel Commun Signal 14 (2), 193–205. 10.1007/s12079-020-00550-x PMC7272539 32067207

[B51] Wang S. Wu Y. Yang S. Liu X. Lu Y. Liu F. (2020b). miR-874 Directly Targets AQP3 to Inhibit Cell Proliferation, Mobility and EMT in Non-small Cell Lung Cancer. Thorac. Cancer 11 (6), 1550–1558. 10.1111/1759-7714.13428 32301290 PMC7262918

[B52] Wang W. Li J. Ding Z. Li Y. Wang J. Chen S. (2019). Tanshinone I Inhibits the Growth and Metastasis of Osteosarcoma via Suppressing JAK/STAT3 Signalling Pathway. J. Cel Mol Med 23 (9), 6454–6465. 10.1111/jcmm.14539 PMC6714145 31293090

[B53] Wang Y. Chen H. Wei X. (2021). Circ_0007142 Downregulates miR-874-3p-Mediated GDPD5 on Colorectal Cancer Cells. Eur. J. Clin. Invest. 51 (7), e13541. 10.1111/eci.13541 33797091

[B54] Wei Y. Wang Z. Wei L. Li S. Qiu X. Liu C. (2021). MicroRNA-874-3p Promotes Testosterone-Induced Granulosa Cell Apoptosis by Suppressing HDAC1-Mediated P53 Deacetylation. Exp. Ther. Med. 21 (4), 359. 10.3892/etm.2021.9790 33732332 PMC7903439

[B55] Witek L. Janikowski T. Gabriel I. Bodzek P. Olejek A. (2021). Analysis of microRNA Regulating Cell Cycle-Related Tumor Suppressor Genes in Endometrial Cancer Patients. Hum. Cel 34 (2), 564–569. 10.1007/s13577-020-00451-6 PMC7900021 33123872

[B56] Witt O. Deubzer H. E. Milde T. Oehme I. (2009). HDAC Family: What Are the Cancer Relevant Targets? Cancer Lett. 277 (1), 8–21. 10.1016/j.canlet.2008.08.016 18824292

[B57] Wu G. Zhou H. Li D. Zhi Y. Liu Y. Li J. (2020). LncRNA DANCR Upregulation Induced by TUFT1 Promotes Malignant Progression in Triple Negative Breast Cancer via miR-874-3p-SOX2 axis. Exp. Cel Res 396 (2), 112331. 10.1016/j.yexcr.2020.112331 33058834

[B58] Xia B. Lin M. Dong W. Chen H. Li B. Zhang X. (2018). Upregulation of miR-874-3p and miR-874-5p Inhibits Epithelial Ovarian Cancer Malignancy via SIK2. J. Biochem. Mol. Toxicol. 32 (8), e22168. 10.1002/jbt.22168 30004169

[B59] Xie K. Cai Y. Yang P. Du F. Wu K. (2020). Upregulating microRNA-874-3p Inhibits CXCL12 Expression to Promote Angiogenesis and Suppress Inflammatory Response in Ischemic Stroke. Am. J. Physiol. Cel Physiol 319 (3), C579–C588. 10.1152/ajpcell.00001.2020 32608990

[B60] Yan Y. Song X. Li Z. Zhang J. Ren J. Wu J. (2017). Elevated Levels of Granzyme B Correlated with miR-874-3p Downregulation in Patients with Acute Myocardial Infarction. Biomark Med. 11 (9), 761–767. 10.2217/bmm-2017-0144 28699362

[B61] Yang H. Dong Y. Zhou Y. Li H. (2021). Overexpression of miR-874-3p Alleviates LPS-Induced Apoptosis and Inflammation in Alveolar Epithelial Cell by Targeting EGR3/NF-kappaB. Acta Biochim. Pol. 68 (2), 231–238. 10.18388/abp.2020_5523 34038062

[B62] Yang R. Li P. Zhang G. Lu C. Wang H. Zhao G. (2017). Long Non-coding RNA XLOC_008466 Functions as an Oncogene in Human Non-small Cell Lung Cancer by Targeting miR-874. Cell Physiol Biochem 42 (1), 126–136. 10.1159/000477121 28501870

[B63] Yao H. Ashihara E. Maekawa T. (2011). Targeting the Wnt/beta-Catenin Signaling Pathway in Human Cancers. Expert Opin. Ther. Targets 15 (7), 873–887. 10.1517/14728222.2011.577418 21486121

[B64] Yao T. Zha D. Gao P. Shui H. Wu X. (2018). MiR-874 Alleviates Renal Injury and Inflammatory Response in Diabetic Nephropathy through Targeting Toll-like Receptor-4. J. Cel Physiol 234 (1), 871–879. 10.1002/jcp.26908 30171701

[B65] Yu C. Li S. Hu X. (2020). Circ_0005576 Promotes Malignant Progression through miR-874/CDK8 Axis in Colorectal Cancer. Onco Targets Ther. 13, 7793–7805. 10.2147/OTT.S249494 32848415 PMC7430768

[B66] Yuan F. Zhao Z. T. Jia B. Wang Y. P. Lei W. (2020). TSN Inhibits Cell Proliferation, Migration, Invasion, and EMT through Regulating miR-874/HMGB2/beta-Catenin Pathway in Gastric Cancer. Neoplasma 67 (5), 1012–1021. 10.4149/neo_2020_190919N931 32484696

[B67] Yuan R. B. Zhang S. H. He Y. Zhang X. Y. Zhang Y. B. (2018). MiR-874-3p Is an Independent Prognostic Factor and Functions as an Anti-oncomir in Esophageal Squamous Cell Carcinoma via Targeting STAT3. Eur. Rev. Med. Pharmacol. Sci. 22 (21), 7265–7273. 10.26355/eurrev_201811_16261 30468470

[B68] Zhang H. Liu L. Chen L. Liu H. Ren S. Tao Y. (2021a). Long Noncoding RNA DANCR Confers Cytarabine Resistance in Acute Myeloid Leukemia by Activating Autophagy via the miR-874-3P/ATG16L1 axis. Mol. Oncol. 15 (4), 1203–1216. 10.1002/1878-0261.12661 33638615 PMC8024725

[B69] Zhang L. Yan D. L. Yang F. Wang D. D. Chen X. Wu J. Z. (2017). DNA Methylation Mediated Silencing of microRNA-874 Is a Promising Diagnosis and Prognostic Marker in Breast Cancer. Oncotarget 8 (28), 45496–45505. 10.18632/oncotarget.17569 28525377 PMC5542203

[B70] Zhang S. Zhang Y. Wang N. Wang Y. Nie H. Zhang Y. (2021b). Long Non-coding RNA MIAT Impairs Neurological Function in Ischemic Stroke via Up-Regulating microRNA-874-3p-Targeted IL1B. Brain Res. Bull. 175, 81–89. 10.1016/j.brainresbull.2021.07.005 34265390

[B71] Zhang X. Tang J. Zhi X. Xie K. Wang W. Li Z. (2015). miR-874 Functions as a Tumor Suppressor by Inhibiting Angiogenesis through STAT3/VEGF-A Pathway in Gastric Cancer. Oncotarget 6 (3), 1605–1617. 10.18632/oncotarget.2748 25596740 PMC4359318

[B72] Zhang Y. Wang X. Zhao Y. (2018a). MicroRNA874 Prohibits the Proliferation and Invasion of Retinoblastoma Cells by Directly Targeting Metadherin. Mol. Med. Rep. 18 (3), 3099–3105. 10.3892/mmr.2018.9295 30015932

[B73] Zhang Y. Wei Y. Li X. Liang X. Wang L. Song J. (2018b). microRNA-874 Suppresses Tumor Proliferation and Metastasis in Hepatocellular Carcinoma by Targeting the DOR/EGFR/ERK Pathway. Cel Death Dis 9 (2), 130. 10.1038/s41419-017-0131-3 PMC5833540 29374140

[B74] Zhang Z. Yang W. Li N. Chen X. Ma F. Yang J. (2021c). LncRNA MCF2L-AS1 Aggravates Proliferation, Invasion and Glycolysis of Colorectal Cancer Cells via the Crosstalk with miR-874-3p/FOXM1 Signaling axis. Carcinogenesis 42 (2), 263–271. 10.1093/carcin/bgaa093 32860508

[B75] Zhao B. Dong A. S. (2016). MiR-874 Inhibits Cell Growth and Induces Apoptosis by Targeting STAT3 in Human Colorectal Cancer Cells. Eur. Rev. Med. Pharmacol. Sci. 20 (2), 269–277. 26875895

[B76] Zhao J. Li H. Chang N. (2020). LncRNA HOTAIR Promotes MPP+-Induced Neuronal Injury in Parkinson's Disease by Regulating the miR-874-5p/ATG10 axis. EXCLI J. 19, 1141–1153. 10.17179/excli2020-2286 33013268 PMC7527508

